# Functional Analysis of RSS Spacers

**DOI:** 10.1371/journal.pbio.0000004

**Published:** 2003-10-13

Based on sheer numbers, microbes should rule the world. Most don't cause disease, but those that do have the advantage of multiplying and mutating at a much faster rate than any multicellular organism can. So how does a slowly reproducing, trillion-celled organism like a human protect itself? By having the right weapon for the job—and that requires an incredibly diverse arsenal. A new study by a team of researchers from Yale University School of Medicine, Duke University Medical Center, and Mount Sinai School of Medicine demonstrates how the creation of that arsenal depends on a complex series of interactions between key genetic elements and proteins during the formation of the white blood cells called lymphocytes.

Two heavy hitters of the immune system—B and T cells—each produce unique protein receptors that specifically recognize and mediate the killing of the variety of potential foreign invaders, or antigens, such as bacteria, viruses, and parasites. (B cells make immunoglobulin, or antibodies, and T cells make T-cell receptors.) But these lymphocytes are unlike other cells: instead of making proteins from genes they inherited, they custom-make their genes by recombining fragments of their genes into new configurations. This genetic reshuffling process, called V(D)J recombination, yields the diversity of molecules necessary to combat the billions of different antigens they might encounter. The V, D, and J refer to different clusters of DNA sequences that follow specific rules of recombination.

While the products of recombination vary, the method does not. The fragments are spliced and then reassembled in a highly regulated process directed and controlled by a stretch of DNA (called a recombination signal sequence, or RSS) next to the gene fragment. The recombination process, the researchers show, relies on complex interactions among different parts of the signal sequences and the proteins that regulate them at key steps along the recombination pathway.

Each RSS is made up of three components: the nonamer, which controls the ability of proteins to bind to the gene fragments and initiate recombination; the heptamer, which directs the splicing of the gene fragment; and the spacer, which regulates how the gene fragments are recombined. Mutations in the DNA sequence of each of the three RSS components show that all play a critical role in the ability of the gene fragments to recombine appropriately.

While it has been established that spacers, as their name suggests, ensure that the space between the nonamer and heptamer is correct, the researchers show that spacers also regulate recombination activity by providing protein-binding sites along the DNA sequences that affect recombination. While the nonamer is the most important determinant of recombination, changes in the spacer, these researchers demonstrate, produced dramatic changes in the ability of the gene fragments to recombine.

Past studies have shown that recombination depends on the presence and sequence of specific nucleotides, but the quality of that recombination, the researchers say, can't be understood simply by analyzing those nucleotides in isolation. Generally speaking, highly conserved sequences have functional importance. But it would be a mistake, they suggest, to think that just because a nucleotide sequence isn't highly conserved, it's not biologically important. Using a computer model to predict how different protein-gene interactions affect recombination, the researchers demonstrate that a fuller understanding of the process depends on observing how all these elements—including those that aren't highly conserved—interact throughout the recombination process.

